# A systematic review of economic evaluations of health and health-related interventions in Bangladesh

**DOI:** 10.1186/1478-7547-9-12

**Published:** 2011-07-20

**Authors:** Mohammad E Hoque, Jahangir AM Khan, Shahed SA Hossain, Rukhsana Gazi, Harun-ar Rashid, Tracey P Koehlmoos, Damian G Walker

**Affiliations:** 1 Health system and Economics Unit, ICDDR,B: Center for Health and Population Research, GPO Box 128, Dhaka-1000, Bangladesh; 2 Bangladesh Medical Research Council, Dhaka, Bangladesh; 3 Financial and Health Policy, Global Health program, Bill and Melinda Gates Foundation, Seattle, USA

## Abstract

**Background:**

Economic evaluation is used for effective resource allocation in health sector. Accumulated knowledge about economic evaluation of health programs in Bangladesh is not currently available. While a number of economic evaluation studies have been performed in Bangladesh, no systematic investigation of the studies has been done to our knowledge. The aim of this current study is to systematically review the published articles in peer-reviewed journals on economic evaluation of health and health-related interventions in Bangladesh.

**Methods:**

Literature searches was carried out during November-December 2008 with a combination of key words, MeSH terms and other free text terms as suitable for the purpose. A comprehensive search strategy was developed to search Medline by the PubMed interface. The first specific interest was mapping the articles considering the areas of exploration by economic evaluation and the second interest was to scrutiny the methodological quality of studies. The methodological quality of economic evaluation of all articles has been scrutinized against the checklist developed by Evers Silvia and associates.

**Result:**

Of 1784 potential articles 12 were accepted for inclusion. Ten studies described the competing alternatives clearly and only two articles stated the perspective of their articles clearly. All studies included direct cost, incurred by the providers. Only one study included the cost of community donated resources and volunteer costs. Two studies calculated the incremental cost effectiveness ratio (ICER). Six of the studies applied some sort of sensitivity analysis. Two of the studies discussed financial affordability of expected implementers and four studies discussed the issue of generalizability for application in different context.

**Conclusion:**

Very few economic evaluation studies in Bangladesh are found in different areas of health and health-related interventions, which does not provide a strong basis of knowledge in the area. The most frequently applied economic evaluation is cost-effectiveness analysis. The majority of the studies did not follow the scientific method of economic evaluation process, which consequently resulted into lack of robustness of the analyses. Capacity building on economic evaluation of health and health-related programs should be enhanced.

## Background

Resource scarcity is a common reality in the health sectors of low income countries. Given that the allocation and identification of additional resources is a major political decision and a long-term planning issue for government, many countries concentrate on more effectively utilizing the available resources instead. One method used for priority setting among health interventions is economic evaluation [1]. Though a number of economic evaluation studies in health sector have been carried out in Bangladesh, it is not clear whether the Bangladeshi policy makers utilize economic evaluation evidence in resource allocation decisions or setting priorities in achieving health coverage goals. It is also debatable, as in other low and middle income countries; among the policy makers of Bangladesh whether it is appropriate and feasible to introduce economic evaluation data into the health care priority making decisions.

Systematic reviews of economic evaluation studies have been carried out in various settings and in different areas of interest [2-8]. While Damian and Fox-Rushby [3] reviewed economic evaluation targeting communicable diseases, Mills and Thomas [2] concentrated on health programs in developing countries. Our interest concerns an investigation regarding the state of art of economic evaluation research in Bangladesh. Country-specific investigations have been carried out earlier in other countries including Thailand [7] and Australia [8].

In previous literature reviews in this area, the authors indicated a number of shortcomings in the published literature. In developing countries limited local capacity in undertaking economic evaluations and failure to monitor the quality of the studies has been observed [3]. Experience from developed countries show that methods used in economic evaluation is extremely heterogeneous and applied in an ad hoc basis [9-13].

While a number of economic evaluation studies have been performed in Bangladesh, no systematic investigation on which intervention areas are explored by economic evaluation and the quality of the studies have not been done to our knowledge. The articles on economic evaluation of health and health-related interventions in Bangladesh will be scrutinized on the basis of a checklist, developed by Evers et al [14].

Evers et al [14] published an article titled, "Criteria list for assessment of methodological quality of economic evaluations: Consensus on Health Economic Criteria" based on an outcome of the project "Consensus on Health Economic Criteria (CHEC)". Under this project, the authors developed a criteria list for assessment of the methodological quality of economic evaluations in systematic reviews. The criteria list was produced through employing a Delphi method including three Delphi rounds for reaching consensus among twenty-three international experts in the panel. A consensus over a generic core set of items for the quality assessment of economic evaluations was achieved among the experts. Each item of the CHEC list was then formulated as a question for answering either by "yes" or "no". The project team, in addition, provided an operationalization of the criteria list items to standardize the interpretation of the list and to make it user-friendly. This checklist can be used for making the future systematic reviews of economic evaluations more transparent, informative, and comparable. The criteria mentioned in the checklist are given in table 1.

The aim of this study is to systematically review the published literature on economic evaluation of health and health-related interventions in Bangladesh. The first specific interest is to map the articles by subject area under economic evaluation and the second interest is to assess the methodological quality of these studies.

## Methods

### Search Strategy

A comprehensive search strategy was developed to search Medline via the PubMed interface. The search was limited to all publications indexed from January 1, 1971 to December 30, 2008. The literature search was carried out during December 2008 with a combination of key words, MeSH terms and other free text terms as suitable for the purpose. The full search strategy is available in Additional file 1.

In addition we also searched minor databases such as Eldis, WHOLIS, World Bank, USAID, Management Sciences for Health (MSH), DFID and Centre for Reviews and Dissemination (CRD) database hosted at York University, and Google scholar. We also undertook hand searching of reference lists of relevant papers and reviews identified. However, the PubMed search covered all other search results.

### Inclusion Criteria

This study set out to identify and include all published articles that included an economic evaluation of health and related interventions in Bangladesh. We considered studies that used primary or secondary data. We limited our search to studies published in English language and related to humans.

### Exclusion criteria

We excluded studies not conducted in humans, not in the health sector, and not in Bangladesh. Studies were excluded if they do not present any kind of cost or expenditure related data, or if they were editorial, review or methodological articles.

## Results

This section is presented in three parts: the results of the search staretgy, a mapping of the economic evaluation literature and a review of the technical characteristics of the articles. With the mapping part, the main interest is to explore in which areas economic evaluation research has been done. Secondly, in the technical characteristics part, the interest is to observe if the reviewed studies have followed the methodological quality of economic evaluation.

### I. Search results

**Figure 1:**
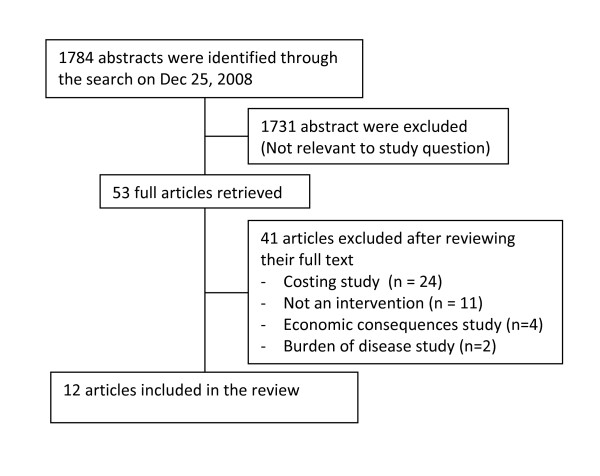
**Flowchart of study selection process**.

A total of 1784 abstract were identified from the search done in December, 2008. Two reviewers screened the abstracts individually and excluded 1731 titles and/or abstracts. Fifty-three full text articles were retrieved. After a second round of double screening, 12 articles were judged to be eligible for inclusion in the review (Additional file 2). More information about the 41 excluded full text articles appears in the table of excluded studies (Additional file 3). Two reviewers then conducted data abstraction. See Figure 1 for a flow chart of study selection process.

### II. Mapping the articles

The number of articles on economic evaluation of healthcare programs in Bangladesh, published in international journals is very limited and there is considerable heterogeneity. Areas of variation include date of publication, the subject of evaluation, and the methods used for evaluation. Only 12 articles have been published in last three decades. In 1980s, only one article has been published. However, the number increased to four in 1990s and seven since 2000. A quick overview of all reviewed articles, containing information on author's affiliation, collaborator, funding agency, type of economic evaluation, and categories (disease or program specific) of studies in a matrix form is presented in additional file 4.

Though the number of articles increased over decades, a very few number of articles in total has been published during our period of investigation (1971-2008). The first article based on economic evaluation of health and health-related intervention in Bangladesh was published in year 1983 though our search period starts from 1971. Only, two studies (Reference number 7 and 8 in additional file 4) have been carried out by authors (first) affiliated with any institution located in Bangladesh; however, each study included collaboration with Bangladeshi institutions.

The subject matter of economic evaluation studies vary largely. Three of the studies dealt with disease-specific economic evaluations (Reference number 1, 7 and 12 in additional file 4) while nine consider health programs. Alternative interventions against diarrhea, tuberculosis and vaccination against measles, yellow fever, BCG, DTP-hep B are analyzed in the disease-specific ones. Among the program-specific ones, there are studies on family planning, maternal service, parasite control, education for awareness and behavior change communication.

Out of the twelve articles, eight articles revealed their funding sources. Of these eight articles, two were supported by the Bill and Melinda Gates Foundation and two by the World Bank. Sources of funding for other three studies are Save the Children - UK, USAID, Sasakawa Health Science Foundation, and DFID. Only one study was supported partially by a domestic funding agency, BRAC.

Three types of economic evaluation have been carried out in the published articles. The most frequently found economic evaluation is cost-effectiveness analysis (10 studies). We found only one cost-minimization analysis and one cost-utility analysis.

### III. Technical characteristics

The methodological quality of economic evaluation of all articles has been assessed against the checklist developed by Evers Silvia et al [14]. Table 1 shows the extent to which the twelve included studies meet the recommendations for good reporting of economic evaluations.

**Table 1:** Methodological Assessment of Economic Evaluations in Bangladesh

Criteria	Yes	No
Description of competitive alternative	10	2
Well defined questions in answerable form	12	0
Economic evaluation study design appropriately	11	1
Time horizon	12	0
Perspective	11	1
All cost item included	9	3
All cost measured appropriately	9	3
All costs valued properly	12	0
Base year of cost data stated	9	3
Sources of cost data included	11	1
Sources of outcome data included	9	3
ICER done	2	10
Cost discounted	3	9
Outcome discounted	2	10
Sensitivity analysis done	6	6
Conclusion follow from the data reported	12	0
Discussed about generasilibity	3	9
Ethical issue discussed	4	8
Affordability discussed	1	11

Ten studies described the competing alternatives clearly. The two studies (Reference number 9 and 12) which did not describe the alternatives clearly, tried to compare the programs with the do-nothing alternative, though it was not explicitly mentioned in the article. All studies posed a study question in answerable form and designed the economic study appropriately. All of the articles considered time horizon in their analyses and the time period ranged between one to seven years.

Considering the perspective of the economic evaluation is important as this determines which costs and effects should be incorporated in the study. Only two authors stated the perspective of their articles clearly. However, after reviewing the articles, the perspective of the studies could be understood, though not explicitly mentioned. Some of the studies considered societal perspective. Such studies included cost components like costs (salary) of health workers, capital cost, recurrent cost, training cost and cost borne by patients like, household out-of-pocket expenditure. However, there are some studies which considered provider's perspective.

Any economic evaluation of health intervention should identify the costs incurred in accordance with intervention alternatives. All studies included direct cost, incurred by the providers. Nine articles included all major costs, such as, personnel cost, capital cost, recurrent cost. One article (reference number 1), which explicitly mentioned its perspective from a provider's point of view, did not include some important cost items, like, administrative and logistic costs. Another study (reference number 3), which took the societal perspective, did not include household cost and staff cost. Only one study (reference number 10) included the cost of community donated resources and volunteer costs, i.e., community donated time. All articles, except one with reference number 3 in additional file 4, informed about the sources of cost data. However, in many cases, these data sources or the procedure of data collection were not clearly described. Levin A (2007) and Goldie SJ (2008) collected data solely from secondary sources, whereas the rest of the studies used data from both primary and secondary sources within the same study. The various inclusive techniques of data collection are employed in the studies: observation and interviews of the health staffs (3 articles), record review, report or literature review (10 articles), patient interview or survey (4 articles) and price-list review (1 article).

Measuring the cost data in appropriate physical unit is important. Only two studies (reference number 6 and 7) used the ingredient approach for stepwise resource allocation. These studies apportioned the joint or overhead cost. Nine studies used discounting for lifetime adjustment of capital and recurrent costs. Four studies (reference number 1, 3, 6 and 7) used the shared cost of health staffs to measure the percentage of time devoted by health workers. In most of the cases, the calculation of cost components is not clearly described. Capital costs, such as building and equipments; recurrent cost, like food, transport, medical supplies etc were not applied in a systematic manner. Nine studies stated the base year of the cost data. The currency used for cost valuation includes US dollars (5 studies), international dollar by one study (reference number 12) and local currency (6 studies).

Most of the studies measured the outcomes using natural units, like proportion of patients cured, share of children immunized etc. Three of the studies employed health outcome as a measure of intervention effect. Two of these studies (reference number 4 and 6) used quality adjusted life years (QALYs) and one used (reference number 12) disability adjusted life years (DALYs) as outcome measurement. Most of the studies measured multiple outcomes of same intervention. The other outcome measurements, not mutually exclusive in the articles, used are patient cured (2 studies), knowledge improvement (3 studies), reduction in prevalence rate of worms (1 study), number of children immunized (2 studies), achieving 80% weight for height (1 study). Nine studies stated the sources of outcome data and multiple sources were used for collecting such information. In six of the studies, the authors implemented intervention programs and created outcome data in comparison with control groups. In other studies, secondary data sources have been used through reviewing published data or literature and estimation by Meta-analysis.

Discounting has been applied in few studies. Costs have been discounted in three (reference number 2, 7 and 11) and outcomes in two studies (reference number 11 and 12). In these studies either 3% or 5% discount rate was employed for costs. The outcome discounting rate was 3%. Two of the studies (reference number 11 and 12) referred to previous studies as a justification for considering the discounting rate, applied in their studies.

Only two studies (reference number 11 and 12) calculated the incremental cost effectiveness ratio (ICER). For calculating ICER, these studies calculated the incremental cost per DALY averted. Both the studies that calculated ICER have compared their results with a benchmark ceiling rate. One study (reference number 11) used the World Bank ceiling ratio of cost per DALY averted which is $175 and the other study (reference number 12) used a ceiling ratio as I$ 29/DALY averted. However, one more study (reference number 6) mentioned that the ICER is important to be calculated, though not applied in that current study.

Performing sensitivity analysis is vital to assess the robustness of the results to changes in assumptions and values of inputs. Six of the studies applied some sort of sensitivity analysis. Five of the studies performed one way sensitivity analysis considering uncertainty of single component (like, upper and lower estimation of QALY-gained) of economic evaluation. Only one study (reference number 10) applied sensitivity analyses applying changes in three different scenarios, namely lower estimate of effectiveness, full costs of implementation and worst-case scenario, i.e. combination of lower effectiveness and full cost.

Two of the studies discussed affordability (reference number 7 and 12), of which one mentioned that 50% more patients can be treated using the existing budget which indicated the affordability of cost-effective intervention, whereas the second one referred to a real world budget constraint. Four studies discussed the issue of generalizability and three of those (reference number 5, 6 and 9) mentioned that the findings of the studies can be replicable in different contexts. One study (reference number 6), on the contrary, proposed for testing in other countries to determine if the intervention is replicable elsewhere. Ethical and distribution issues are not discussed appropriately in most of the articles. However, the study by Taylor M (2003) referred to ethical consideration of collecting data (reference number 9).

## Discussion

From the review of articles, we found that the research-base for economic evaluation of health and health-related interventions in Bangladesh is weak and the studies carried out in this area have many limitations.

According to the mapping of the articles, we found few articles on economic evaluation of health and health-related intervention programs. At the same time these articles addressed a wide number of intervention areas. Thus there is shallow knowledge in a wide number of areas, with no single area or type of intervention being fully investigated. For the use of evidence as a basis for policy making, the same areas should be independently investigated by several research teams. However, this level of investment in economic evaluation should be supported by the use of these studies in health sector decision making.

The contribution of the researchers from Bangladesh-based organizations appears negligible in the broader body of economic evaluation literature. In most of the articles, Bangladeshi researchers appeared as collaborative partners, not the principal investigator or first author. While appearing as a collaborator, their contribution to the research paper is not clearly described. A better understanding of the use and methodology of economic evaluation might help to enhance the translation of knowledge generated by economic evaluations in health sector decision making in Bangladesh.

We have found that among the economic evaluation studies cost-effectiveness analysis is highly prevalent. This can be due to the availability of information on effectiveness in natural terms (like, patients treated, number of visits, persons vaccinated etc.) from the programs and application of straight-forward methods in such evaluations. It has been further observed that although a number of studies were designed for performing CEA, the final analyses of many of these studies were done by comparing the cost and effectiveness ratio, which finally turned into a cost outcome study. Cost-utility analysis, on the other hand, is less frequently found, probably due to the relative difficulty and resource consumption needed for measuring health status, and in the consideration of quality and disability. However, we observed that the concepts of 'cost-effectiveness analysis' and 'cost-utility analysis' are often used interchangeably by the authors. Mislabeling the cost-minimization analysis (CMA) as the CEA was found in the review. The study by Ashworth A (1997) was designed as a CEA; the study used a fixed value for effectiveness measurement which is actually a CMA. Again, cost-benefit analysis for evaluating health and health-related interventions has not been carried out in Bangladesh.

The economic evaluation studies, we reviewed are mainly limited to intervention programs. Several untouched areas can be identified such as disease-specific treatment alternatives, alternative drugs etc.

**Figure 2:**
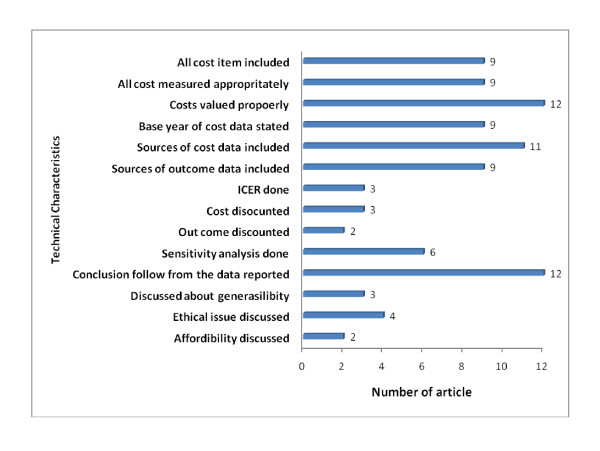
**Number of articles that addressed the criteria of a good economic evaluation according to the checklist**.

The technical characteristics of the included studies show many limitations. Few published economic evaluations have consistently followed correct analytic procedure. In the figure below, we present how many of the 12 articles followed the criteria of being a scientifically good economic evaluation although the articles due a better job of meeting less technical issues. For instance, description of comparative intervention alternatives, appropriateness of study design and perspective taken are completed or at least addressed by most of the articles. Inclusion of all cost items, its measurement and valuing, which are fundamental and more technical issue in economic evaluation, is done by most of the articles. It indicates that the researchers are more familiar with costing techniques. On the other hand, more sophisticated issues, like incremental cost-effectiveness ratio, discounting of costs and effectiveness, sensitivity analysis, discussion on generasibility, ethical issues and affordability were not addressed by most of the articles. It needs to be emphasized here that though less technical issues are addressed by most of the articles; there are many shortcomings in their presentation. For instance, comparative alternatives, though described in the articles, but not in a structured way in many of them. See Figure 2 for a list of articles that addressed the criteria of a good economic evaluation according to the checklist.

Perspective of economic evaluation is an important issue which determines measurements of costs and outcomes of the interventions under investigation. In many studies, we found that though perspective was not clearly stated in some articles, all the studies had a perspective which could be found by reviewing. In some cases, all costs items have not been identified, which underestimates the actual costs of intervention. Traditionally, the studied did not estimated costs of donated items and volunteers' times which are very frequently applicable in a developing country context. However, exclusion of any important cost component can be due to lack of proper training about measurement techniques. Moreover, there is a lack of guideline for costing method with a common consensus of researchers and relevant expertise which can address the standard methodological as well as practical issues in Bangladesh.

Irrespective of kinds of economic evaluation, the reviewed articles are routinely display a lack of transparency. In some cases it is difficult to understand which alternatives are being compared. We even observed when comparing a new intervention or alternative against no intervention, it has not been clearly mentioned in the article. Lack of transparency is found in using cost data from secondary sources. Though the cost and outcome data sources are mentioned in many articles, they mostly lacked a detail description of data collecting process by the sources. In addition, cost from secondary sources (especially, if from other countries) needs to be converted by an appropriate exchange rate. In that case, price inflation over time and purchasing power parity (PPP) between countries needs to be considered. Some studies used US dollar rate, without considering the variation in purchasing power between Bangladesh and USA. PPP adjusted dollar rate needs to be applied instead of simple dollar exchange rate. Furthermore, discounting of costs and outcomes is not used often which consequently provides a biased estimation of economic evaluation. Incremental cost-effectiveness ratio calculation is often missing, which is fundamental for ranking the alternatives while making decision on effective resource allocation.

To validate a study result, the researchers should conduct carry out a sensitivity analysis and triangulation of information. Most of the studies have done sensitivity analyses using only one dimension (like, by changing discount rate). However, for a better validation of the result multi-dimensional sensitivity analyses as well as uncertainty analyses should be done. Triangulation of results can be done by verifying with other relevant studies elsewhere.

Most of the studies lack a discussion on generalisibility of the results, ethical consideration and affordability of the intervention programs. Since the intervention programs often are carried out on a pilot basis in a specific context, a discussion on generalisibility is important for scaling up the programs on the basis of the findings. If the interventions can be done without violating the ethical recommendation, should be clearly discussed. The distribution of outcomes of intervention programs across socioeconomic, age and gender groups etc. need to be discussed as well. Though an intervention program is cost-effective, it may not be affordable in a specific society. For fitting the economic evaluation study in a practical context, affordability of the intervention alternatives should be discussed.

The economic evaluation is an aid to decision making, the quality of published work needs to be improved to ensure that the economic evaluations do not mislead decision makers. The weaknesses in health economic evaluation in Bangladesh may be due to lack of training on economic evaluation methods among the non-economist investigators and shortage of trained health economist in this area of research in Bangladesh. A country specific costing guideline with common consensus among the local researcher and relevant expertise is important for doing a economic evaluation of various health program. A uniform methodological guideline for conducting economic evaluation in Bangladesh is also needed.

Economic evaluation should be focused on interventions that have major impact on population health. There are lack of economic evaluation studies in Bangladesh targeting MDGs, mainly Goal 4 and 5. Various public health programs in maternal and child health should be economically evaluated for scaling up through the country. This is needed to achieve the MDGs by 2015. None of the studies conducted economic evaluation targeting child health and maternal heath has been found. Various economic evaluation studies can be done targeting the national burden of disease, possible alternative interventions and investment in health.

There are some limitations in this current review article. The study searched only the published literature in peer reviewed journals and included literature published in English only. Various gray literature items such as unpublished reports, conference proceedings and reports were not included in the search results. It is possible that more economic evaluation were done by Bangladeshi researchers which are not included here due to the specific inclusion and exclusion criteria.

## Conclusion

In this review we discovered that there are a very few economic evaluations of health interventions. There are many unexplored area such as economic evaluation of disease treatments and alternative drugs. However, one economic evaluation method (cost-effectiveness analysis) was applied most frequently. The majority of the studies did not follow the scientific method of economic evaluation process, which consequently resulted into a lack of robustness of the analyses.

Based on the review of health economic evaluation articles, we recommend that capacity building on economic evaluation of health and health-related programs as well as health economics should be enhanced. Researchers and health sector stakeholders including policy makers and donors should identify important areas of economic evaluation considering national burden of diseases, possible alternative interventions and investment in health. Under the context of limited resources for health in Bangladesh and other developing countries, the use of robust economic evaluation of health and health-related interventions and programmes should be conducted routinely to help guide considerations for generalizability and potential scaling up.

## Competing interests

The authors declare that they have no competing interests.

## Authors' contributions

MEH was involved in concept and design, search strategy, data extraction, designing checklist, data analysis, interpretation of the data, preparing drafts and the manuscript. JK was involved in the analysis, interpreting the data and preparing drafts. SH was involved in conception and design, preparing and run the search strategy and retrieval of the abstract. RG was involved in data extraction, screening and adjuration full text, developing data extraction tool. HR was involved in conception and design and drafting of the manuscript. TK was involved in concept and design, developing data extraction tool and design and drafting of the manuscript. DW was involved in concept and design, search strategy, data extraction, designing checklist and overall guidance of the manuscript. All authors read and approved the final version of the manuscript.

## Supplementary Material

Additional file 1 **Search Strategy**. Click here for file

Additional file 2 **List of selected articles for systematic review**. Click here for file

Additional file 3 **Studies meet the inclusion criteria but are later deemed unsuitable for inclusion**. Click here for file

Additional file 4 **Mapping of included studies**. Click here for file
