# Supplementary material for: A systematic review of economic evaluations of health and health-related interventions in Bangladesh
Source: Cost Eff Resour Alloc. 2011 Jul 20;9:12. doi: 10.1186/1478-7547-9-12 (PMC3158529; doi:10.1186/1478-7547-9-12)
Supplement: Additional file 1 — Search Strategy. [file 1478-7547-9-12-S1.DOC]

**Search strategy**

Databases: Medline/PubMed

Search time: December, 2008

Total Number of hits: 1784

1. Health care economics and organizations[All Fields]
2. (econom* OR cost* OR costing OR price* OR pricing[tiab])
3. pharmacoeconomic[tiab]
4. (expenditure* NOT energy[tiab])
5. (health care cost[tiab]) OR (budget[tiab]) OR ("cost AND cost analysis") OR ("cost estimation"[tiab]) OR ("cost effective"[tiab]) OR ("cost effectiveness"[tiab]) OR ("cost study"[tiab]) OR ("cost studies"[tiab]) OR ("cost calculation"[tiab]) OR ("cost benefit"[tiab]) OR ("cost utility"[tiab]) OR ("cost allocation"[tiab]) OR ("cost control"[tiab]) OR ("cost saving"[tiab]) OR ("cost sharing"[tiab]) OR ("cost appraisal") OR ("cost of illness"[tiab])
6. ("DALY")) OR ("disability adjusted life year*")
7. ("quality adjusted life year*") OR ("QALY")
8. ("economic appraisal"[tiab]) OR ("economic analysis"[tiab]) OR ("economic evaluation*"[tiab])
9. OR /1-8
10. Bangladesh [All fields]
11. # 9 AND # 10
12. Limits: English Language, Publications between January 1971 to December 2008, Humans only
