# Supplementary material for: A systematic review of economic evaluations of health and health-related interventions in Bangladesh
Source: Cost Eff Resour Alloc. 2011 Jul 20;9:12. doi: 10.1186/1478-7547-9-12 (PMC3158529; doi:10.1186/1478-7547-9-12)
Supplement: Additional file 2 — List of selected articles for systematic review. [file 1478-7547-9-12-S2.DOC]

**List of selected articles for systematic review**

1. Ashworth A, Khanum S: **Cost-effective treatment for severely malnourished children: what is the best approach?** *Health Policy and Planning* 1997, **12(2)**: 115-121.
2. Goldie SJ, Diaz M, Kim SY, Levin CE, Van MV, Kim JJ: **Mathematical Models of Cervical Cancer Prevention in the Asia Pacific Region**. *Vaccine* 2008, **26S**: M17–M29
3. Horton S, Claquin P: **Cost-effectiveness and user characteristics of clinic based services for the treatment of Diarrhea: A case study in Bangladesh**. *Social Science and Medicine* 1983, **17(11)**: 721-729.
4. Hutchinson P, Lance P, Guilkey D, Shahjahan M, Haque S: **Measuring the cost-effectiveness of a national health communications program in rural Bangladesh**. *Journal of Health Communication* 2006, **11(Suppl. 2)**, 91–121.
5. Islam MA, Wakai S, Ishikawa N, Chowdhury AMR, Vaughan JP: **Cost-effectiveness of community health workers in tuberculosis control in Bangladesh**. *Bulletin of the World Health Organization* 2002, **80**: 445-450.
6. Khan MM, Ahmed S: **Relative efficiency of government and non-government**

**organisations in implementing a nutrition intervention programme – a case study from Bangladesh.** *Public Health Nutrition* 2003, **6(1):** 19–24**.**

1. Levin A, Amin A, Rahman A, Saifi R. Khuda BE, Mozumder K: **Cost- effectiveness of family planning and maternal health service delivery stretagies in rural Bangladesh**. *International Journal of Health Planning and Management* 1999, **14**: 219-233.
2. Levin A, Levin C, Kristensen D, Matthias D: **An economic evaluation of thermostable vaccines in Cambodia, Ghana and Bangladesh**. *Vaccine* 2007, **25**: 6945–6957.
3. Mascie-Taylor CGN, Alam M, Montanari RM, Karim R, Ahmed T, Karim E, Akhtar S: **A study of the cost effectiveness of selective health interventions for the control of intestinal parasites in rural Bangladesh**. *Journal of Parasitology* 1999, **85(1)**: 6-11.
4. Mascie-Taylor CGN, Karim R, Karim E, Akhtar S, Ahmed T, Montanari RM: **The cost- effectiveness of health education in improving knowledge and awareness about intestinal parasites in rural Bangladesh**. *Economics and Human Biology* 2003, **1(3)**: 21–330.
5. Routh S, Khuda BE. **An economic appraisal of alternative strategies for the delivery of MCH-FP services in Urban Dhaka, Bangladesh**. *International Journal of Health Planning and Management* 2000, **15**:115-132.
6. Simmons GB, Balk D, Faiz KK: **Cost-effectiveness analysis of family planning programs in rural Bangladesh: Evidence from Matlab**. **Studies in Family Planning** 1991, **22(2)**: 83-101.
