# Supplementary material for: A systematic review of economic evaluations of health and health-related interventions in Bangladesh
Source: Cost Eff Resour Alloc. 2011 Jul 20;9:12. doi: 10.1186/1478-7547-9-12 (PMC3158529; doi:10.1186/1478-7547-9-12)
Supplement: Additional file 3 — Studies meet the inclusion criteria but are later deemed unsuitable for inclusion. [file 1478-7547-9-12-S3.DOC]

**Studies meet the inclusion criteria but are later deemed unsuitable for inclusion.**

1. Afsana K. **The tremendous cost of seeking hospital obstetric care in Bangladesh.** *Reprod Health Matters* 2004, **12(24)**: 171-80.
2. Aftabuddin M, Islam N, et al: **Management of isolated radial or ulnar arteries at the forearm**. *J Trauma* 1995, **38(1)**: 149-51.
3. Amin M, Hanson K, et al: **Price discrimination in obstetric services--a case study in Bangladesh**. *Health Econ* 2004, **13(6)**: 597-604.
4. Anoopa Sharma D, Bern C et al: **The economic impact of visceral leishmaniasis on households in Bangladesh.** *Trop Med Int Health* 2006, **11(5)**: 757-64.
5. Borghi J, Sabina N, et al: **Household costs of healthcare during pregnancy, delivery, and the postpartum period: a case study from Matlab, Bangladesh**. *J Health Popul Nutr* 2006, **24(4)**: 446-55.
6. Brown LV, Rogers BL et al: **Comparison of the costs of compliance with nutrition education messages to improve the diets of Bangladeshi breastfeeding mothers and weaning-age children,** 1993 *Ecol Food Nutr* **30(2)**: 99-126.
7. Chawdhury FA, Sultana J et al: **Evaluation of goat blood as substitute for sheep blood in Modified Thayer-Martin agar medium for culture and isolation of Neisseria gonorrhoeae** 2006 *Sex Transm Dis* **33(3)**: 181-2.
8. Chowdhury AM, Karim F et al: **Teaching ORT to women: individually or in groups?** *J Trop Med Hyg* 1988, **91(6)**: 283-7.
9. Chowdhury Z: **The mother and child in Bangladesh. A view from the People's Health Centre (Gonoshasthaya Kendra)** 1976, *Assignment Child* **33**: 68-77.
10. Croft RA and Croft RP (1998): **Expenditure and loss of income incurred by tuberculosis patients before reaching effective treatment in Bangladesh.** *Int J Tuberc Lung Dis* 1976, **2(3)**: 252-4.
11. Ensor T, Ali L et al: **Projecting the cost of essential services in Bangladesh.** *Int J Health Plann Manage* 2003, **18(2)**: 137-49.
12. Fewtrell L, Fuge R et al: **An estimation of the global burden of disease due to skin lesions caused by arsenic in drinking water.** *J Water Health* 2005, **3(2)**: 101-7.
13. Fiedler JL and Day LM: **A cost analysis of family planning in Bangladesh.** *Int J Health Plann Manage* 1997, **12(4)**: 251-77.
14. Finger WR: **Cost analysis plays vital role.** *Network* 1995, **16(1)**: 9-13.
15. Finger WR: **Cost analysis serves many purposes**. *Network* 1998, 18(2): 16-9.
16. Gazi R, Mercer A et al: **Effectiveness of depot-holders introduced in urban areas: evidence from a pilot in Bangladesh**. *J Health Popul Nutr*, 2005, 23(4): 377-87.
17. Heinzen RR and Bridges JF: **Comparison of four contingent valuation methods to estimate the economic value of a pneumococcal vaccine in Bangladesh**. *Int J Technol Assess Health Care* 2008, **24(4)**: 481-7.
18. Hossain MI, Wahed MA et al: **Increased food intake after the addition of amylase-rich flour to supplementary food for malnourished children in rural communities of Bangladesh**. *Food Nutr Bull* 2005, **26(4)**: 323-9.
19. Islam MA, Mahalanabis D et al: **Use of rice-based oral rehydration solution in a large diarrhoea treatment centre in Bangladesh: in-house production, use and relative cost.** *J Trop Med Hyg* 1994, **97(6):** 341-6.
20. Joya SA, Mostofa G et al: **One solution to the arsenic problem: a return to surface (improved dug) wells.** *J Health Popul Nutr* 2006, **24(3)**: 363-75.
21. Kay BJ and. Kabir SM: **A study of costs and behavioral outcomes of menstrual regulation services in Bangladesh.** *Soc Sci Med* 1988, **26(6)**: 597-604.
22. Khan AR, Begum SF et al: **Risks and costs of illegally induced abortion in Bangladesh.** *J Biosoc Sci* 1984, **16(1)**: 89-98.
23. Khan MM, Ali D et al: **A cost-minimization approach to planning the geographical distribution of health facilities.** *Health Policy Plan* 2001, **16(3)**: 264-72.
24. Khan MM, Khan SH et al: **Cost of delivering child immunization services in urban Bangladesh: a study based on facility-level surveys.** *J Health Popul Nutr* 2004, **22(4)**: 404-12.
25. Khan MM, Saha KK et al: **Adopting integrated management of childhood illness module at local level in Bangladesh: implications for recurrent costs**. *J Health Popul Nutr* 2002, **20(1)**: 42-50.
26. Khan SH: **Free does not mean affordable: maternity patient expenditures in a public hospital in Bangladesh.** *Cost Eff Resour Alloc* 2005, **3(1)**: 1.
27. Kibriya MG, Ali L et al: **Home monitoring of blood glucose (HMBG) in Type-2 diabetes mellitus in a developing country.** *Diabetes Res Clin Pract* 1999, **46(3)**: 253-7.
28. Killingsworth JR, Hossain N et al: **Unofficial fees in Bangladesh: price, equity and institutional issues.** *Health Policy Plan* 1999, **14(2)**: 152-63.
29. Lokuge KM, Smith W et al: **The effect of arsenic mitigation interventions on disease burden in Bangladesh.** *Environ Health Perspect* 2004, **112(11)**: 1172-7.
30. Mashreky SR, Rahman A et al: **Burn injury: economic and social impact on a family.** *Public Health* 2008, **122(12)**: 1418-24.
31. Mashreky SR, Rahman A et al: **Consequences of childhood burn: findings from the largest community-based injury survey in Bangladesh.** *Burns* 2008, **34(7)**: 912-8.
32. McCord C. and Chowdhury Q: **A cost effective small hospital in Bangladesh: what it can mean for emergency obstetric care**. *Int J Gynaecol Obstet* 2003, **81(1)**: 83-92.
33. Nahar S and Costello A. **The hidden cost of 'free' maternity care in Dhaka, Bangladesh.** *Health Policy Plan* 1998, **13(4)**: 417-22.
34. Oberle MW, Merson MH et al: **Diarrhoeal disease in Bangladesh: epidemiology, mortality averted and costs at a rural treatment centre.** *Int J Epidemiol* 1980, **9(4)**: 341-8.
35. Routh SA, Thwin A et al: **Cost efficiency in maternal and child health and family planning service delivery in Bangladesh: implications for NGOs.** *Health Policy Plan* 2004, **19(1)**: 11-21.
36. Salam MA, Noguchi T et al: **Factors affecting participating farmers' willingness-to-pay for the Tree Farming Fund: a study in a participatory forest in Bangladesh.** *Environ Monit Assess* 2006, **118(1-3)**: 165-78.
37. Stanton B and Clemens J: **User fees for health care in developing countries: a case study of Bangladesh.** *Soc Sci Med* 1989, 29(10): 1199-205.
38. Ur Rashid H: **Health delivery system for renal disease care in Bangladesh.** *Saudi J Kidney Dis Transpl* 2004, **15(2)**: 185-9.
39. Van Doorslaer E, O'Donnell O et al: **Catastrophic payments for health care in Asia.** *Health Econ* 2007, **16(11)**: 1159-84.
40. Van Doorslaer, E, O'Donnell O et al: **Effect of payments for health care on poverty estimates in 11 countries in Asia: an analysis of household survey data**. *Lancet* 2006, **368(9544)**: 1357-64.
41. Zakir Hussain AM: **Cost analysis of a primary health care centre in Bangladesh.** *Bull World Health Organ* 1983, **61(3)**: 477-83.
