# Supplementary material for: A systematic review of economic evaluations of health and health-related interventions in Bangladesh
Source: Cost Eff Resour Alloc. 2011 Jul 20;9:12. doi: 10.1186/1478-7547-9-12 (PMC3158529; doi:10.1186/1478-7547-9-12)
Supplement: Additional file 4 — Mapping of included studies. [file 1478-7547-9-12-S4.DOC]

Mapping of included studies

| **Reference number** | **Name of the article** | **Article reference** | **Journal** | **First author affiliation** | **Collaborator** | **Funding agency** | **Type of EE** | **Category** | |
| --- | --- | --- | --- | --- | --- | --- | --- | --- | --- |
|  |  |  |  |  |  |  |  | Disease-specific | Program-specific |
| 1. | Cost-effectiveness and user characteristics of clinic based services for the treatment of diarrhea: a case study in Bangladesh | Horton S. 1983 | Social Science and Medicine | University of Toronto, Canada | ICDDR,B | Not mentioned | CEA | Diarrohea |  |
| 2. | Cost-effectiveness analysis of family planning programs in rural Bangladesh: Evidence from Matlab | Simmons GB. 1991 | Studies in Family Planning | University of Michigan, USA | ICDDR,B | Not mentioned | CEA |  | Family planning programs |
| 3. | Cost effectiveness treatment for severely malnourished children: what is the best approach? | Ashworth A. 1997 | Health Policy and Planning | LSHTM, UK | LSHTM | Save the children Fund, UK | CMA |  | In-patient management |
| 4. | Cost- Effectiveness of Family Planning and Maternal Health Service Delivery Strategies in Rural Bangladesh | Levin A. 1999 | Int J Health Planning and Management | PHR University Research Corporation, USA | ICDDR,B | Bill & Melinda Gates Foundation | CEA |  | FP-MHS |
| 5. | A study of the cost effectiveness of selective health interventions for the control of intestinal parasites in rural Bangladesh | Taylor M. 1999 | J Parasitology | University of Cambridge, UK | Dhaka University IEDCR, Dhaka WHO, Dhaka | World Bank Consortium | CEA |  | Health interventions for parasites control |
| 6. | An economic appraisal of alternative strategies for the delivery of MCH-FP services in urban Dhaka, Bangladesh. | Routh S. 2000 | Int J Health Planning Management | ICDDR,B  Bangladesh | None | USAID | CUA |  | MCH-FP services |
| 7. | Cost Effectiveness of community health workers in tuberculosis control in Bangladesh | Islam MA. 2002 | Bulleting of WHO | BRAC, Bangladesh | University of Tokyo; RIT Japan; LSHTM | Sasakawa Health Foundation, RIT Japan, BRAC | CEA | TB control |  |
| 8. | Relative efficiency of government and non-government organizations in implementing a nutrition intervention program – a case study from Bangladesh | Khan MM. 2003 | Public Health Nutrition | Tulane University School of Public Health, New Orleans, LA, USA | ICDDR,B | Department for International Development (UK) | CEA |  | Nutrition intervention program |
| 9. | The cost effectiveness of health education in improving knowledge and awareness about intestinal parasite in rural Bangladesh | Taylor M. 2003 | Economics and Human Biology | University of Cambridge, UK | IPHN, Dhaka University IEDCR, WHO, Dhaka | World Bank Consortium | CEA |  | Knowledge and awareness about parasite |
| 10. | Measuring the cost-effectiveness of a national health communication program in rural Bangladesh | Hutchinson P. 2006 | Journal of Health Communication | Tulane University, USA | BCCP, Bangladesh | Not mentioned | CEA |  | Communication program |
| 11. | An economic evaluation of thermos table vaccines in Cambodia, Ghana and Bangladesh | Levin A. 2007 | Vaccine | PATH, USA | None | Not mentioned | CEA | Vaccine products |  |
| 12. | Mathematical Models of Cervical Cancer Prevention in the Asia Pacific Region | Goldie S. 2008 | Vaccine | Harvard SPH, USA | Harvard, USA  CERDP, Spain  PATH, USA  Hanoi Medical University, Vietnam | Bill and Melinda Gates Foundation | CEA | Vaccination & screening |  |
